# Supplementary figures and images for: Seroprevalence and incidence of hepatitis A in Southeast Asia: A systematic review
Source: PLoS One. 2021 Dec 1;16(12):e0258659. doi: 10.1371/journal.pone.0258659 (PMC8635355; doi:10.1371/journal.pone.0258659)

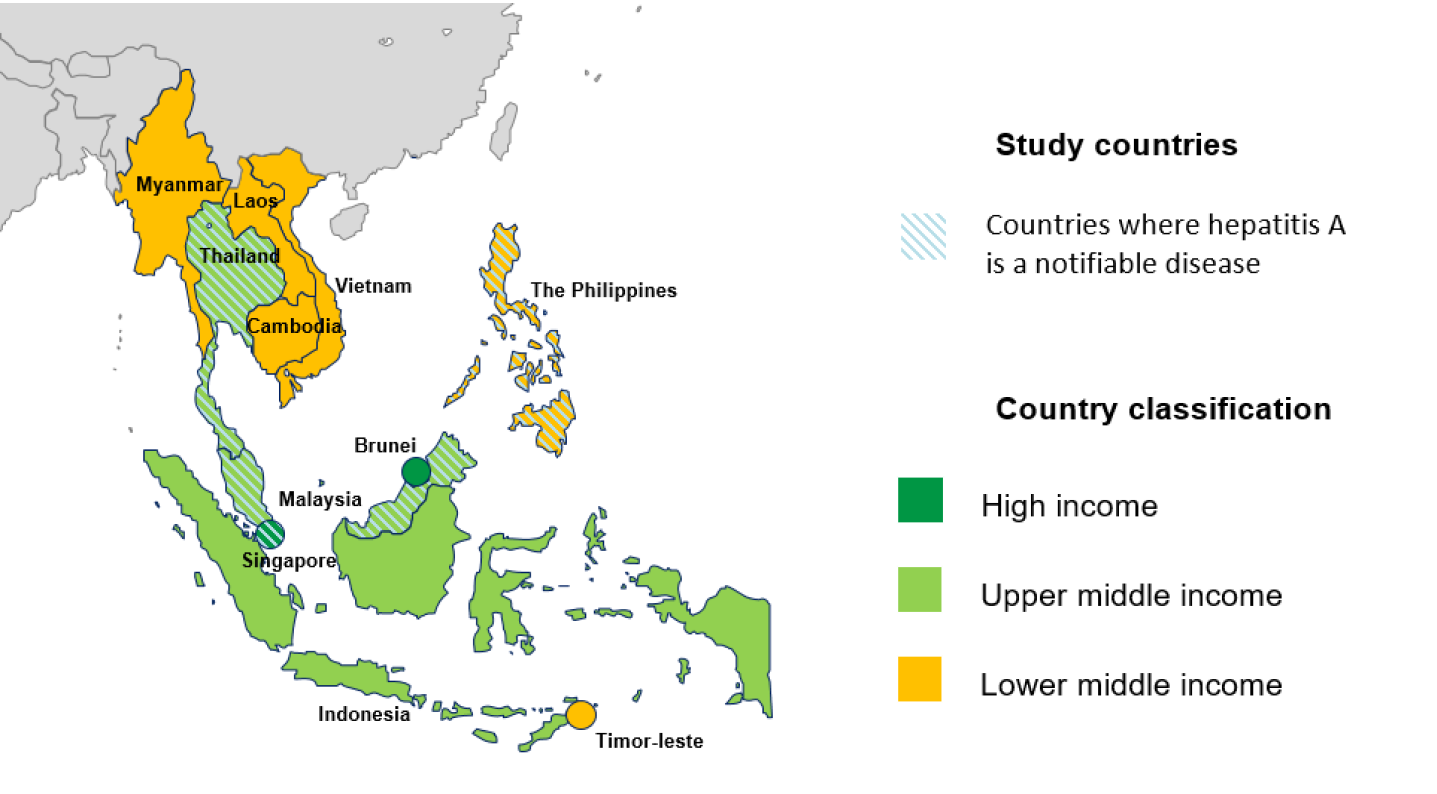

Supplement: S1 Fig — (TIFF) [file pone.0258659.s002.tiff]
