# Supplementary material for: Seroprevalence and incidence of hepatitis A in Southeast Asia: A systematic review
Source: PLoS One. 2021 Dec 1;16(12):e0258659. doi: 10.1371/journal.pone.0258659 (PMC8635355; doi:10.1371/journal.pone.0258659)
Supplement: S1 Text — (DOCX) [file pone.0258659.s003.docx]

**[S1 Text:** Search Strategy]

We developed a broad search strategy using free-text terms (hepatitis A epidemiology, incidence, prevalence, morbidity, mortality) linked by Boolean operators with indexed terms. For PubMed, we utilized medical subject heading (MeSH). The search syntax in PubMed was (("asia, southeastern"[MeSH Terms] AND ("Prevalence"[MeSH Terms] AND "Hepatitis A"[All Fields])) OR ("Hepatitis"[MeSH Major Topic] AND (("Brunei"[MeSH Terms] OR "Cambodia"[MeSH Terms] OR "Indonesia"[MeSH Terms] OR "Laos"[MeSH Terms] OR "Malaysia"[MeSH Terms] OR "Myanmar"[MeSH Terms] OR "Philippines"[MeSH Terms] OR "Singapore"[MeSH Terms] OR "Thailand"[MeSH Terms] OR "Timor-Leste"[MeSH Terms]) AND "Vietnam"[MeSH Terms])) OR ("Hepatitis A"[MeSH Terms] AND "asia, southeastern"[MeSH Terms]) OR ("Seroepidemiologic Studies"[MeSH Terms] AND "Hepatitis"[All Fields] AND "asia, southeastern"[MeSH Terms]) OR ((("Hepatitis A"[MeSH Terms] AND "asia, southeastern"[MeSH Terms]) OR ("Seroepidemiologic Studies"[MeSH Terms] AND "Hepatitis"[MeSH Terms] AND "asia, southeastern"[MeSH Terms])) AND 1999/01/01:2021/02/15[Date - Publication]) OR ("asia, southeastern"[MeSH Terms] AND ("incidence"[MeSH Terms] AND "Hepatitis"[MeSH Terms]) AND 1999/01/01:2021/02/15[Date - Publication]))
