# Supplementary material for: Seroprevalence and incidence of hepatitis A in Southeast Asia: A systematic review
Source: PLoS One. 2021 Dec 1;16(12):e0258659. doi: 10.1371/journal.pone.0258659 (PMC8635355; doi:10.1371/journal.pone.0258659)
Supplement: S1 Table — *References cited by screened articles were manually reviewed for relevance (i.e. snowballing). **References of included articles in these systematic reviews/meta-analyses were manually screened for additional relevant original articles (as deemed necessary by the reviewer).Abbreviations: HAV, hepatitis A virus; n.a., not applicable. (DOCX) [file pone.0258659.s004.docx]

**Supplementary table 1**: Inclusion and exclusion criteria

|  | **Inclusion criteria** | **Exclusion criteria** |
| --- | --- | --- |
| Population | - Hepatitis A disease (not limited to risk groups or specific ages) | - Populations with chronic diseases or underlying comorbidities that are not representative of the general population |
| Main exposure | - Age group | - n.a. |
| Comparator | - n.a. | - n.a. |
| Outcome | - HAV seroprevalence - HAV incidence | - n.a. |
| Study design | - Primary peer-reviewed research*   - Observational studies     - Cohort studies     - Case-control studies     - Cross-sectional studies     - Ecological studies     - Outbreak investigations     - Periodic surveys   - Non-peer-reviewed research     - Reports from national and regional databases or websites | - Non-primary research   - Systematic reviews**   - Meta-analyses**   - Narrative reviews (without methods) - Predictions via modelling methods - Case reports - Letter to editor - Newspaper - Editorial - Comment - Opinions |
| **Limits** | | |
| Publication date | - From January 01, 1999 until February 15, 2021 | - |
| Geographic scope | - Brunei - Cambodia - Indonesia - Lao PDR - Malaysia - Myanmar - the Philippines - Singapore - Thailand - Timor-Leste - Vietnam | - |
| Language | - English - Local languages:   - Burmese   - Cambodian   - Indonesian   - Lao PDR   - Malay   - Filipino   - Thai   - Vietnamese | - |

*References cited by screened articles were manually reviewed for relevance (i.e. snowballing)

**References of included articles in these systematic reviews/meta-analyses were manually screened for additional relevant original articles (as deemed necessary by the reviewer).

Abbreviations: HAV, hepatitis A virus; n.a, not applicable
